# Vigilance-Avoidance Toward Negative Faces in Social Anxiety With and Without Comorbid Depression

**DOI:** 10.3389/fpsyt.2021.636961

**Published:** 2021-03-31

**Authors:** Tomoko Kishimoto, Xu Wen, Mingzhu Li, Ru-Yuan Zhang, Nisha Yao, Yunzhen Huang, Mingyi Qian

**Affiliations:** ^1^Department of Social Psychology, Zhou Enlai School of Government, Nankai University, Tianjin, China; ^2^Beijing Key Laboratory for Behavior and Mental Health, School of Psychological and Cognitive Sciences, Peking University, Beijing, China; ^3^George Warren Brown School of Social work, Washington University in St. Louis, Missouri, TX, United States; ^4^Shanghai Key Laboratory of Psychotic Disorders, Shanghai Mental Health Center, School of Medicine, Shanghai Jiao Tong University, Shanghai, China; ^5^Institute of Psychology and Behavioral Science, Shanghai Jiao Tong University, Shanghai, China; ^6^State Key Laboratory of Brain and Cognitive Science, Chinese Academy of Sciences (CAS) Center for Excellence in Brain Science and Intelligence Technology, Institute of Psychology, Chinese Academy of Sciences, Beijing, China

**Keywords:** social anxiety disorder, depression, comorbidity, attentional bias, vigilance-avoidance

## Abstract

Despite the growing evidence for the attentional bias toward emotional related stimuli in patients with social anxiety disorder (SAD), it remains unclear how the attentional bias manifests in normal individuals with SAD and/or depressive traits. To address this question, we recruited three groups of normal participants with different psychiatric traits—individuals with comorbid SAD and depression (SADd, *N* = 19), individuals with only SAD (SAD, *N* = 15), and healthy control individuals (HC, *N* = 19). In a dot-probe paradigm, participants view angry, disgusted, and sad face stimuli with durations ranging from very brief (i.e., 14ms) that renders stimuli completely intangible, to relatively long (i.e., 2000ms) that guarantees image visibility. We find significant early vigilance (i.e., on brief stimuli) and later avoidance (i.e., on long stimuli) toward angry faces in the SADd group. We also find vigilance toward angry and disgusted faces in the SAD group. To our best knowledge, this is the first study to unify both vigilance and avoidance within the same experimental paradigm, providing direct evidence for the “vigilance-avoidance” theory of comorbid SAD and depression. In sum, these results provide evidence for the potential behavioral differences induced by anxiety-depression comorbidity and a single trait in non-clinical populations, but the lack of a depression-only group cannot reveal the effects of high levels of depression on the results. The limitations are discussed.

## Introduction

Social anxiety disorder (SAD) is a common debilitating emotional disorder and can cause severe emotional and social dysfunctions (1). One hypothesis is that people with SAD have an abnormally higher sensitivity to socially threatening information. Such aberrant hypersensitivity enhances the degree of subjectively perceived threats, and, as a consequence, hinders social interactions (2). This theory is supported by the lab-based experimental finding that people with SAD exhibit attentional hypersensitivity to socially threatening stimuli, such as negative emotional faces (3–5).

**Figure 1:**
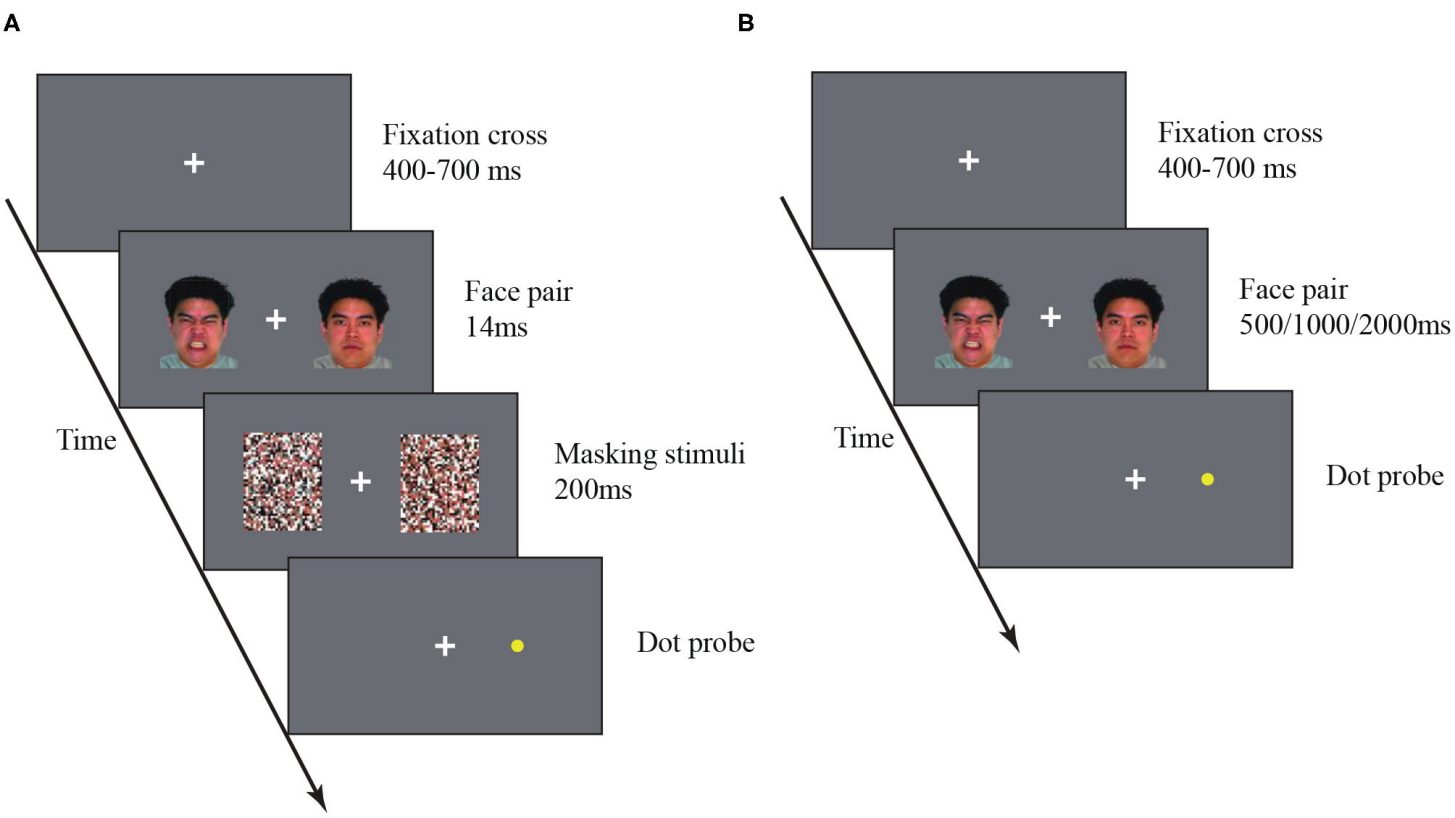
Flow diagram of the subliminal **(A)** and supraliminal **(B)** dot-probe experiments.

One frequently used task in this domain is the dot-probe task. In this task, an emotional visual stimulus and a neutral stimulus are simultaneously presented on the two sides of a computer screen. Visual masks may be included to eliminate visual afterimage. A dot probe then appears at one side, and the subject is asked to report the location of the dot probe as fast and accurately as possible (Figure 1). If the emotional stimulus indeed attracts attention, the subject will respond faster and more accurately to the probe dot that appears on the same side of the emotional stimulus. Using this paradigm, researchers not only discover the attentional bias in SAD but also find that stimulus duration mediates the strength of the attentional bias. For example, SAD individuals show attentional preferences to anxiety-related words (3, 4) and faces with negative expressions (5). But this effect only holds for brief stimuli (e.g., <500ms) (6) not for longer stimuli (7). Interestingly, other evidence shows SAD individuals also avoid paying attention to the stimuli with longer durations (e.g., 1000ms) (8, 9). These results prompt a new “vigilance-avoidance” theory that socially threatening information evokes a fast initial attentional attraction followed by a late attentional avoidance in people with SAD (10). However, the phenomena of attentional preference and avoidance have been investigated mostly in distinct studies. These studies employed different experimental settings, such as stimulus duration and types. It remains unclear whether the two perceptual signatures of SAD arise from an independent or unified mechanism.

Despite the sizeable evidence of atypical attentional processing in SAD people, one underexplored issue is how their perceptual deficits are modulated by other comorbid psychiatric traits, especially depression. This question is particularly important for several reasons. First, the strong comorbidity of SAD and depression have been well-established in clinical populations (11–13). Second, individuals with depression also exhibit strong attentional bias. But their bias is toward mood-congruent/negative emotional stimuli, such as sad faces (14–16). The comorbidity with depression may alter SAD people's attentional bias. Third, there is existing evidence that people with comorbid SAD and depression (SADd) have more severe symptoms, poorer social functioning, and a stronger tendency of clinical prognosis compared with people with only one disorder (17, 18). These findings suggest a shared pathological basis of SAD and depression. Lastly, although atypical attentional behavior has been established in clinical populations, it remains unclear how the comorbidity of SAD and depression manifests in normal populations merely with heightened traits. Non-clinical samples have been used in several previous studies (4). Epidemiological data shows that social anxiety is a continuous existence (19), SAD has no clear threshold, and as social fear increases, the degree of injury increases linearly (20). Examinations on those who show a tendency toward SAD and depression may provide useful insights into early detection and prevention of the diseases.

Compared to the investigations conducted independently on SAD and depression, only a few attempts have been made to investigate the attentional bias in SADd. Given the severer clinical symptoms of SADd, one may predict a stronger attentional bias in SADd patients as compared to patients with only one disorder. Existing results, however, are controversial. For example, one study found that comorbid depression seemed to attenuate the attentional bias in SADd as compared to individuals with only SAD (3). This stands in contrast to other studies reporting a similar level of attentional preferences in both SADd and SAD patients (16, 21).

We surmise that the diverse stimulus durations and stimulus types used in previous studies lead to ostensibly conflicting results. As aforementioned, the attentional bias in SAD strongly depends on stimulus duration (22–24). Furthermore, the examination of SAD and depression requires distinct emotion-congruent stimuli, such as angry faces for SAD and sad faces for depression. Most previous studies only employed a limited set of stimulus conditions, which may lead to inconsistent behavioral patterns (7, 25, 26).

The aims of this study are three-fold. First, we aim to compare the attentional bias related to the SADd traits and the SAD traits. Second, we focus on healthy participants with psychiatric traits rather than clinical samples in order to reveal behavioral signatures in sub-clinical populations. Third, to reconcile conflicting results in previous studies, we plan to systematically manipulate stimulus duration and stimulus type (5, 14, 21). In addition to duration conditions used in previous studies, we included 14-ms stimuli that are too brief to be perceivable, triggering unconscious attentional orienting. Comparisons of multiple duration conditions allow us to examine attentional processing of both subliminal and supraliminal stimuli. Consistent with the hypothesized effects of stimulus duration, we predicted that SADd and/or SAD participants would exhibit attentional preferences to the stimuli with short durations and attentional avoidance to those with long durations. We also use sad, disgusted, and angry faces, three basic emotion types related to both anxiety and depression. Angry and disgusting facial expressions are particularly significant for those with social anxiety as they convey important information concerning personal acceptability and social value (7, 27, 28). Additionally, depressed individuals have an attentional bias toward sad faces (29). We hypothesized that SAD-depression commodity enhances sensitivity especially to angry faces for two reasons. First, socially threatening information in angry faces is particularly prominent for both SAD and depression. Second, as mentioned above, the comorbidity with depression may further strengthen the severity of symptoms in SAD.

## Methods and Materials

### Ethics Statement

All experimental protocols were approved by the Ethics Committee of Peking University. All research was performed in accordance with relevant guidelines and regulations. Informed written consent was obtained from all participants.

### Recruitment and Selection of Participants

Before the experiment, we used G^*^Power 3.1.9.6 to estimate the planned sample size (α = 0.05, 1–β = 0.80), and calculated it according to the medium effect size [effect size *f* = 0.25, (30)]. The result of the total sample size was 30, the actual sample size was 52.

Participants were recruited from online platforms of Universities in Beijing. On the platforms, we described the major symptoms of SAD and introduced the recruitment criteria and the purpose of the study (i.e., investigate the cognitive signatures of SAD). We emphasized that this study was irrelevant to SAD treatments, and all volunteers would be paid for participation. A total of 80 participants filled the online questionnaires (see below) and expressed interests in our study. We further selected participants based on the following criteria. The SAD group should have (1) the Social Phobia Scale (SPS) scores above 42 or the Social Interaction Anxiety Scale (SIAS) scores above 52 (31), and (2) the results of the clinical diagnostic scale (CDS, see Materials) should only meet the diagnosis for SAD. The SADd group should have (1) the SPS Scores above 42 or the SIAS scores above 52, and (2) the Beck Depression Inventory (BDI) scores above 15 (32); (3) the results of the CDS should meet the diagnosis for both SAD and depression (including depressive episodes or dysthymia). The HC group should have the SPS scores below 43, the SIAS scores below 53 points, and the BDI scores below 16. No assessments were conducted for other psychiatric disorders. Note that we did not conduct clinical diagnosis interviews, and all selections were based on a clinical diagnostic scale adapted from the Chinese Mini-International Neuropsychiatric Interview (MINI) (see below), because our primary interests were in normal people with psychiatric traits, not clinical samples.

Twenty-two participants were excluded for not meeting the criteria of any group. Nineteen SAD participants, 15 SADd participants, and 24 HC participants met the criteria. We further randomly selected 19 HC participants to participate in our study. All participants were naïve to the purpose of this study. All participants were monolingual native-Chinese speakers, right-handed, and had normal or corrected-to-normal vision.

The three groups were matched in gender [χ^2^(2, N = 53) = 0.21, *p* = 0.902]. Inevitably, there were significant differences in three *pre-test* scales between the groups [SIAS: *F*(2, 50) = 43.98, *p* < 0.001, $\eta_{\text{partial}}^{2}$ = 0.638; SPS: *F*(2, 50) = 15.60, *p* < 0.001, $\eta_{\text{partial}}^{2}$ = 0.384; BDI: *F*(2, 50) = 20.97, *p* < 0.001, $\eta_{\text{partial}}^{2}$ = 0.455]. The detailed demographic information is summarized in the Table 1.

**Table 1:** Demographic information.

	**SAD**	**SADd**	**HC**	***post-hoc* test (LSD)**
N	19	15	19	
Age	23.6 ± 3.6	28.7 ± 5.2	22.2 ± 3.7	➁ > ➀^**^; ➁ > ➂^***^
Female (%)	57.9	60.0	52.6	
SPS	57.37 ± 15.1	64.20 ± 12.8	40.58 ± 10.2	➀ > ➂^***^; ➁ > ➂ ^***^
SIAS	68.68 ± 9.5	69.47 ± 7.7	43.74 ± 10.5	➀ > ➂ ^***^; ➁ > ➂ ^***^
BDI	15.26 ± 8.4	23.87 ± 6.6	8.21 ± 5.7	➀ > ➂ ^**^; ➁ > ➀ ^***^; ➁ > ➂ ^***^

➀, SAD; ➁, SADd; ➂, HC;

*** p ≤ 0.001,

** *p ≤ 0.01*.

### Clinical Assessments

All participants completed the following online questionnaires.

#### Social Interaction Anxiety Scale (SIAS)

SIAS is the most common measure for SAD. It was first created by (33), and the Chinese version consists of 19 items. The scale is scored on a five-point rating scale. The internal consistency is 0.874; the test-retest (after 3 weeks) reliability is 0.863. The internal consistency in this study is 0.923.

#### Social Phobia Scale (SPS)

SPS only measures individuals' fear of negative evaluations in social situations and does not involve other symptoms related to social anxiety, and it is also commonly used in the assessment of SAD. SIAS and SPS are moderately correlated with each other, r = 0.684 (31). SPS was first created by (33), and the Chinese version consists of 20 items. The scale is scored on a five-point rating scale. The internal consistency is 0.862; the test-retest (after 3 weeks) reliability is 0.849. The internal consistency in this study is 0.917.

#### Beck Depression Inventory (BDI)

The BDI was created by Beck (34) and has been used extensively to assess depression. The Chinese version of the BDI was used in this study (32). The scale consists of 21 items, with each item scored from 0 to 3 points. A higher total score indicates more severe depression. This scale has good reliability and validity, and the internal consistency of the Chinese version is 0.890 (32). The internal consistency in this study is 0.891.

#### Clinical Diagnostic Scale (CDS)

A clinical diagnostic scale was adapted from the Chinese Mini-International Neuropsychiatric Interview (MINI) (35). MINI is a short and structured clinical diagnostic interview. MINI diagnoses disorders of Axis I of the DSM-IV by yes/no questions and the questions have good reliability and validity (35). The items measuring depressive episode, dysthymia, and SAD were extracted from the MINI into a self-reported questionnaire. Although the MINI is initially designed for a structured diagnosis interview, we believe it is viable to convert the highly structured MINI to an online self-reported scale because the interview structure is fixed and additional items are not permitted. The self-reported scale mainly focuses on the current symptoms and does not distinguish whether the symptoms are primary or secondary.

### Behavioral Experiments

*Visual stimuli were presented on a Gamma-corrected Iiyama HM204DT 22 inches monitor, with a spatial resolution of 1024* × *768 and a refresh rate of 60 Hz. The viewing distance was about 80 cm. A white cross was always presented at the center of the screen as a fixation, and participants were asked to fixate the cross throughout the experiment*. In addition, in the dot-probe task, participants were asked to use the keyboard to respond. When the probe appears on the left or right side of the screen, the individual responds by pressing the “F” or “J” key, respectively. All participants were required to complete four tasks of stimulus duration, and the order of completion was balanced using Latin square design. All participants completed 10 trials of practice (14ms subliminal stimulation and 500ms supraliminal stimulation) before proceeding to the formal task. After completion, the participants were asked whether they could see the picture clearly when there was a masking stimulus. No participants reported being able to see the picture clearly. If the subjects had no questions about the task, they would enter the formal experiment.

#### Emotional Face Stimuli

The faces of 10 different identities (five male and five female) with four different emotions (angry, sad, disgusted, and neutral) were selected from the NimStim Face Stimulus Set (36). The mask stimuli were generated by scrambling the face images. The size of all face and masking stimuli was 13.5° × 10.8° (visual angle).

#### Subliminal Dot-Probe Task

A white fixation cross was presented at the center of a gray screen, and the participants were asked to stare at the fixation cross throughout the entire experiment. In each trial, the fixation cross was presented for a duration between 400 and 700ms (uniformly distributed). An emotional face and a neutral face were then presented for 14ms on each side (10° eccentricity) of the fixation (14). The locations (i.e., left/right position) of the two face images were randomized across trials. The face images were followed by backward masks for 200ms. A yellow dot-probe then appeared on one side. participants were asked to press a key to indicate on which side the dot-probe appeared. The dot-probe disappeared immediately after a keypress. All participants completed 10 blocks each of 60 trials. In each block, each of the three types of emotional faces were presented over 20 trials. The participants rested for 1 min between two blocks.

#### Supraliminal Dot-Probe Task

The procedure of the supraliminal dot-probe task was the same as the subliminal dot-probe task, except for two distinctions. First, the face images were presented for longer durations (i.e., 500, 1000, and 2000ms) without following masking stimuli (Figure 1B). Second, to avoid fatigue caused by long, continuous tasks, we increase the number of breaks for the 2000ms duration condition, all participants completed 20 blocks each consisting of 30 trials, and the three types of emotional faces were each presented 10 times.

The attentional bias was quantified by the reaction time difference (*D*_*RT*_):

(1) $$\begin{array}{l} D_{RT}=RT_{NF}-RT_{EF}, \end{array}$$

where *RT*_*NF*_ and *RT*_*EF*_ indicate the reaction time to the dot-probes presented after emotional faces and neutral faces, respectively. Positive and negative values of *D*_*RT*_ indicate attention toward (i.e., attentional attraction) or away from (i.e., attentional avoidance) emotional stimuli.

### Handling Outliers

#### Demographic Information

*Two* missing age values were detected and replaced using the mean age of all participants.

#### Dot-Probe Experiment

Outliers in the behavioral data were handled based on the method described by Erceg-Hurn (37, 38), in order to retained most outliers for correct responses. In this method, all trials with wrong responses were discarded and then the difference (interquartile range, *interQ*) between the lower quartile (25%, *)* and the upper quartile (75%, *)* of all correct trials was calculated. The upper limit *L* and lower limit *S* of the reaction time can be calculated using the equations:

(2) $$\begin{array}{lll} L & = & Q2+1.5\times interQ, \end{array}$$

(3) $$\begin{array}{lll} S & = & Q1+1.5\times interQ, \end{array}$$

Finally, all values out of the upper and the lower limit in the correct trials were bounded to the limit values. The preprocessed data were then used in further statistical analyses.

### Statistical Analysis

First, the group differences at the *pre-test* were analyzed. The chi-square test was used to investigate gender differences between the groups. Intergroup differences in age, SIAS, SPS, and BDI were examined by one-way ANOVA, and LSD was used in the *post-hoc* test. Given the significant group differences in age, this was included as a covariable in the subsequent ANOVA analyses. Second, we conducted a three-way repeated-measure ANCOVA, with stimulus duration (14ms/500ms/1500ms/2000ms) and emotion type (anger/disgust/sadness) as the within-group variables, group (SAD/SADd/HC) as the between-group variable, and age as the covariable. Third, because of the significant interaction effect of duration by group by emotion type, three two-way repeated-measure ANOVAs were performed for the three emotion types, with age as the covariate. Pairwise comparisons (using Bonferroni correction) were performed for significant interactions between group and stimulus duration, and Cohen's ds were provided. Further, we conducted 36 *t*-tests (two-tailed) to analyze whether attention bias scores significantly differed from “Zero.”

## Results

### Results of the Three-Way ANCOVA

The sphericity test was not significant (*p* = 0.245). There were no significant main effects of emotion type [*F*(2, 98) = 0.728, *p* = 0.486, $\eta_{\text{partial}}^{2}$ = 0.015], duration [*F*(3, 147) = 0.859, *p* = 0.464, $\eta_{\text{partial}}^{2}$ = 0.017], and group [*F*(2, 49) = 0.581, *p* = 0.563, $\eta_{\text{partial}}^{2}$ = 0.023]. The interaction effect between emotion type and duration was significant [*F*(6, 294) = 2.386, *p* = 0.029, $\eta_{\text{partial}}^{2}$ = 0.046]. However, the subsequent simple effect showed the emotion type differences at each duration, 14ms [*F*(2, 48) = 0.206, *p* = 0.815, $\eta_{\text{partial}}^{2}$ = 0.008], 500ms [*F*(2, 48) = 2.160, *p* = 0.126, $\eta_{\text{partial}}^{2}$ = 0.083], 1000ms [*F*(2, 48) = 1.317, *p* = 0.277, $\eta_{\text{partial}}^{2}$ = 0.052], and 2000ms [*F*(2, 48) = 0.166, *p* = 0.848, $\eta_{\text{partial}}^{2}$ = 0.007] were not significant. The duration by group interaction [*F*(6, 147) = 1.825, *p* = 0.098, $\eta_{\text{partial}}^{2}$ = 0.069] and the emotion by group interaction [*F*(4, 98) = 1.329, *p* = 0.256, $\eta_{\text{partial}}^{2}$ = 0.051] were not significant. The triple interaction among emotion type, duration, and age was significant [*F*(6, 294) = 2.622, *p* = 0.017, $\eta_{\text{partial}}^{2}$ = 0.051]. To further analyze this interaction, we divided the participants into two groups by age (participants with ages under/above the mean age) and conducted a two-way ANOVA separately for each age group. For participants with ages under the mean age (*N* = 33), the main effect of emotion type was significant [*F*(2, 64) = 3.251, *p* = 0.045, $\eta_{\text{partial}}^{2}$ = 0.092], but no significant difference was found in the subsequent pairwise comparisons. The main effect of duration [*F*(3, 96) = 0.017, *p* = 0.997, $\eta_{\text{partial}}^{2}$ = 0.001], and the interaction effect between emotion type and duration [*F*(6, 192) = 1.180, *p* = 0.318, $\eta_{\text{partial}}^{2}$ = 0.036] were not significant. For participants with ages above the mean age (*N* = 20), the main effects of emotion type [*F*(2, 38) = 0.059, *p* = 0.942, $\eta_{\text{partial}}^{2}$ = 0.003] and duration [*F*(3, 57) = 0.638, *p* = 0.191, $\eta_{\text{partial}}^{2}$ = 0.079], and their interaction [*F*(6, 114) = 1.257, *p* = 0.283, $\eta_{\text{partial}}^{2}$ = 0.062] were not significant. These results suggest that age may contribute to the observed attentional bias, but its exact underlying mechanisms are difficult to delineate given the current dataset. Future studies are needed to systematically investigate this issue. The triple interactions among emotion type, duration, and group were significant [*F*(12, 294) = 2.864, *p* = 0.001, $\eta_{\text{partial}}^{2}$ = 0.105]. In the following sections, we separately performed three two-way ANCOVAs to analyze the effects of emotion type and duration in each group.

### Anger

**Figure 2:**
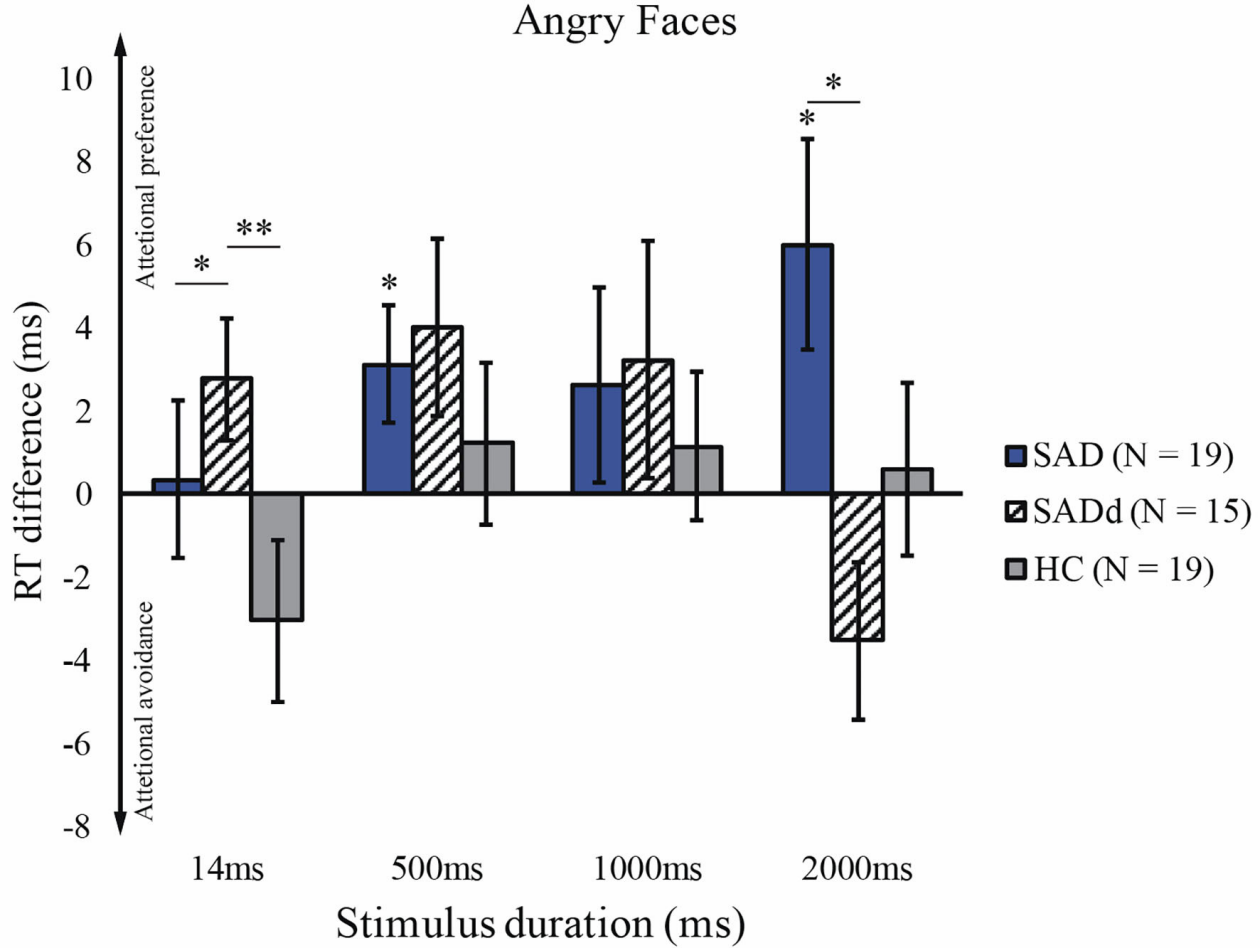
Reaction time difference across groups and stimulus durations for angry faces. The y-axis is the reaction time difference between emotional faces and neutral faces. The error bars represent the S.E.M across participants. Figure conventions are **p < 0.01, *p < 0.05 (two-tailed). These conventions are kept in all subsequent figures.

For the angry faces, we applied the Greenhouse-Geisser correction of ANOVA, given that the sphericity test was significant, *p* = 0.006. Consistent with previous findings, there was a significant main effect of duration [*F*(3, 124) = 5.29, *p* = 0.003, $\eta_{\text{partial}}^{2}$ = 0.097], but no main effect of group [*F*(2, 49) = 2.19, *p* = 0.123, $\eta_{\text{partial}}^{2}$ = 0.082]. However, there was a significant interaction between stimulus duration and group [*F*(5, 124) = 3.20, *p* = 0.009, $\eta_{\text{partial}}^{2}$ = 0.116], and a significant interaction between stimulus duration and age [*F*(3, 124) = 6.16, *p* = 0.001, $\eta_{\text{partial}}^{2}$ = 0.112]. We further tested group differences under each duration using *post-hoc* analysis. We found that the SADd participants had a stronger attentional preference than SAD and HC on subliminal stimuli (SADd vs. SAD: *p* = 0.037, *d* = 0.41; SADd vs. HC: *p* < 0.001, *d* = 0.91). On such a short duration (14 ms), SAD did not show a stronger attentional preference than HC (SAD vs. HC, *p* = 0.131, *d* = 0.53). For 2000ms stimuli, the SADd participants showed a stronger attentional avoidance compared with the attentional preference of SAD participants (*p* = 0.021, *d* = −1.07). No other significant results of the pairwise comparisons were found. The results of *t*-tests showed that SAD did significantly differ from zero at 500 ms (*t*_18_ = 2.17, *p* = 0.044) and 2000ms (*t*_18_ = 2.35, *p* = 0.030). The reaction time differences for the angry faces are presented in Figure 2.

### Disgust

**Figure 3:**
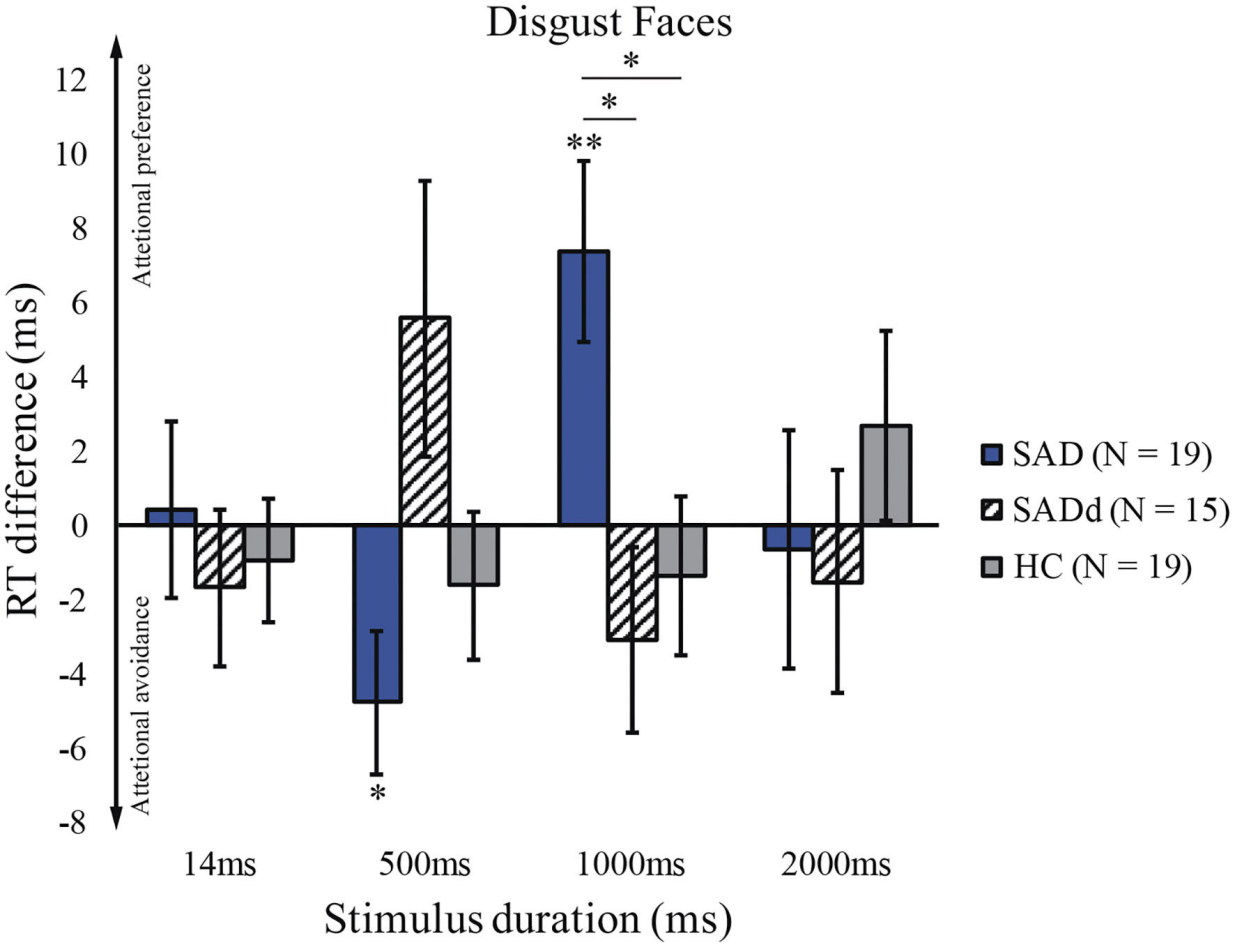
Reaction time difference across groups and stimulus durations for disgusted faces. Figure conventions are **p < 0.01, *p < 0.05 (two-tailed). These conventions are kept in all subsequent figures.

The sphericity test was not significant, *p* = 0.494. For the disgusted faces, we did not find significant main effects of stimulus duration [*F*(3, 147) = 0.468, *p* = 0.705, $\eta_{\text{partial}}^{2}$ = 0.009] and group [*F*(2, 49) = 0.613, *p* = 0.546, $\eta_{\text{partial}}^{2}$ = 0.024]. However, we again found a significant interaction between duration and group [*F*(6,147) = 2.912, *p* = 0.010, $\eta_{\text{partial}}^{2}$ = 0.106]. *Post-hoc* pairwise comparisons showed that the SAD group exhibited a stronger attentional preference than the SADd group (*p* = 0.014, *d* = 1.45) and the HC group (*p* = 0.036, *d* = 1.33) on 1000ms stimuli. No other significant results of pairwise comparisons were detected. The results of the *t*-tests showed that SAD significantly differed from zero at 500ms (*t*_18_ = −2.48, *p* = 0.023) and 1000ms (*t*_18_ = 3.02, *p* = 0.007). The reaction time differences for the disgusted faces are presented in Figure 3.

### Sadness

**Figure 4:**
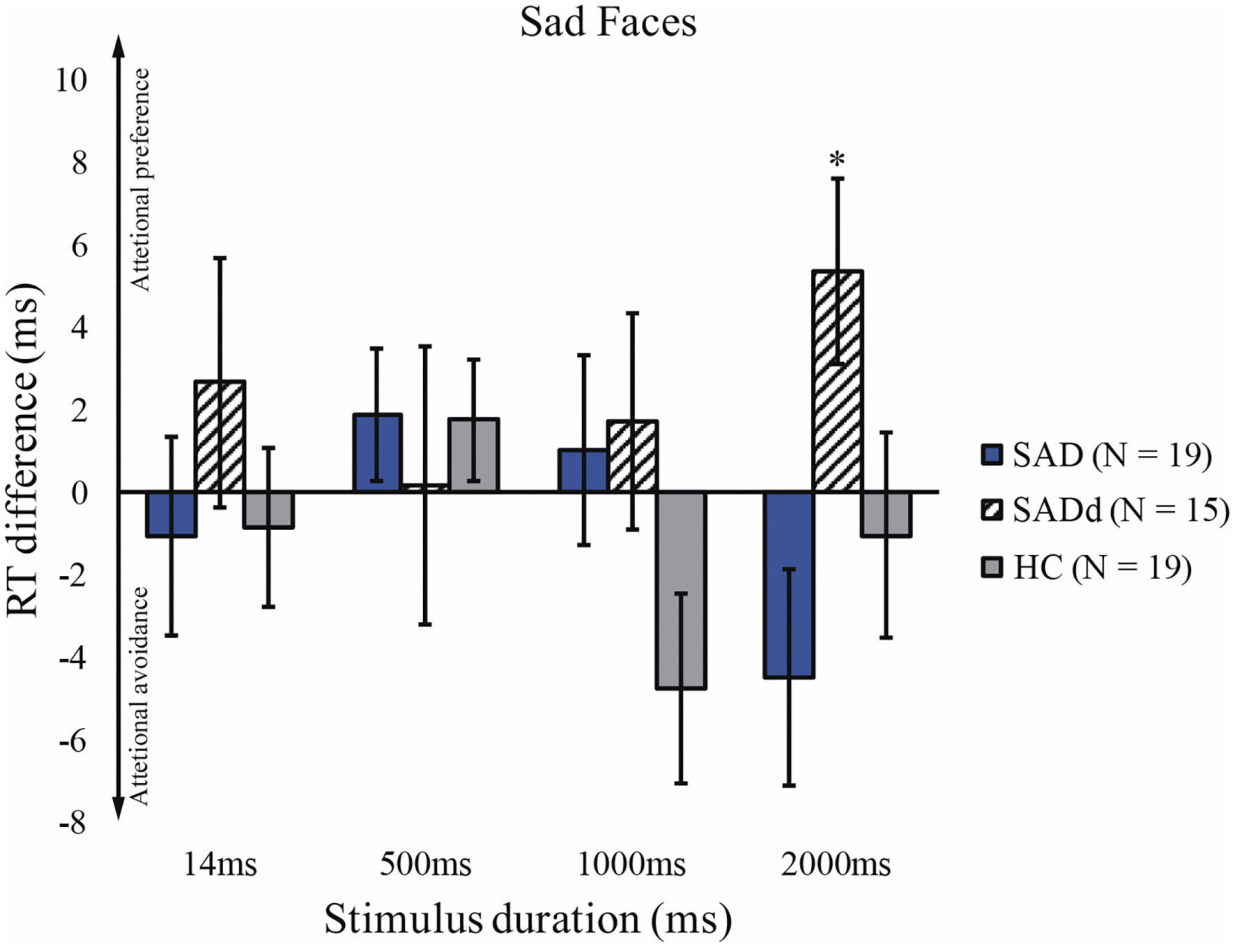
Reaction time differences across groups and stimulus durations for sad faces.

The sphericity test was not significant, *p* = 0.939. For the sad faces, there were no main effects of duration [*F*(3, 147) = 0.749, *p* = 0.525, $\eta_{\text{partial}}^{2}$ = 0.015] and group [*F*(2, 49) = 0.626, *p* = 0.539, $\eta_{\text{partial}}^{2}$ = 0.025]. Moreover, the interaction between duration and group was not significant [*F*(6, 147) = 1.41, *p* = 0.214, $\eta_{\text{partial}}^{2}$ = 0.055]. The result of the *t*-test showed that SADd significantly differed from zero and 2000ms (*t*_14_ = 2.38, *p* = 0.032). The reaction time differences for the sad faces are presented in Figure 4.

## Discussion

In this study, we recruited normal individuals with comorbid SAD and depression traits, and normal individuals with only SAD traits, to investigate the interactive contributions of anxiety and depression traits to attentional bias. Moreover, we systematically manipulated stimulus duration and emotion type, the two key factors that we suspected to cause discrepancies across the literature. We made three major observations. First, SADd participants exhibited a stronger attentional preference for angry faces than SAD and HC participants on short duration (subliminal) stimuli. Second, SADd participants also exhibited a stronger attentional avoidance on longer angry faces (2000ms). Third, for the disgusted faces with durations around 1000ms, SAD participants showed a stronger attentional preference than SADd and HC participants.

Cognitive biases in psychiatric disorders have been an active research area in clinical psychology because perceptual and cognitive deficits may severely impair social behavior and adaptation. However, previous investigations on the attentional bias in SAD are controversial (39). We speculate that uncontrolled stimulus duration and type may explain such discrepancies. For example, (7) only tested 500 ms and 1250ms stimuli. The narrow duration range might not elicit strong attentional bias (6). Only tested the very short duration (i.e., 17ms) and found a significant attentional attraction, which is consistent with our results in SADd groups (40). Only tested 7 and 1000ms stimuli. Here, we used three stimulus types and four stimulus durations, ranging from subliminal and supraliminal stimuli. Most importantly, for the first time, we identified both attentional preference and avoidance within the same experimental paradigm. Interestingly, the preference and the avoidance occur on the stimuli with the shortest (i.e., 14ms) and the longest duration (i.e., 2000ms), respectively. This finding further highlights the importance of using a wider range of stimulus durations to test the attentional bias in SAD.

Stimulus type is another important factor that modulates attentional bias. Different disorders show aberrant sensitivity to diverse stimuli. SAD is especially sensitive to socially threatening information (3, 41). Individuals with depression usually show attentional bias toward mood-congruent negative stimuli, such as sad faces (15). A threatening expression directs some form of hostility at the beholder. Anger is very easily recognizable in other people across a variety of cultures. Disgust bears resemblance to anger because it signals disapproval (41). It has been shown that SAD patients rate disgust stimuli as more negative than angry faces (27). At the neural level, individuals with non-clinical social anxiety do not show differential cortical responses to angry and disgusted faces in a novelty detection task (42). Two studies used the similar dot-probe paradigm and sad and disgusted faces to examine SAD people's attentional bias (4, 43). But they only tested the 500ms condition and did not distinguish angry and disgusted faces and unified them as negative emotions. The two studies indeed found attentional preference toward the negative emotions. Here we found attentional preference toward sad faces but attentional avoidance toward disgusted faces in the SAD group. These discrepancies should be further tested in future work.

Our results also provide evidence for the “vigilance-avoidance” theory. The inconsistent results in prior work prompt the “vigilance-avoidance” theory (19), based on the findings of both attentional preferences and avoidance, depending on stimulus duration (6, 7, 44). To our best knowledge, our study unifies the two effects within the same experimental paradigm and provides the first direct evidence for the “vigilance-avoidance” theory.

What are the contributions of depression to the attentional bias in SAD-depression commodity? Theorists of social anxiety disorder have argued that social fears involving interactions with strangers (45, 46) or authority figures may predict an especially inauspicious course, including risk for comorbid disorders (47). Here, we found both attentional preference and avoidance on angry faces in the SADd group and only attentional preference on disgusted faces in the SAD group. These findings also suggest that apparently conflicting results in previous studies may also be partially explained by not carefully distinguishing the SADd and SAD only subjects. This finding suggests that the inconsistent results in the literature may be partially due to the comorbidity with depression. For example (44), recruited SAD participants who also showed strong depression traits. But in (6, 7), participants were examined only for SAD but may also have had comorbid depression. Our findings here suggest future studies considering the potential effects of comorbid traits when investigating attentional bias. Our results are also of particular significance to the behavioral markers for identifications of clusters of people with a single disorder or psychiatric comorbidity.

It is worth mentioning four potential limitations in this study. First, this study investigated subclinical samples identified by self-report scales. It remains unclear to what extent our results can generalize to inpatient or output patient samples. Direct comparisons between normal individuals with psychiatric traits and formally diagnosed patients are in general an issue in clinical psychology. Only a few studies have shown that the attentional bias in subclinical samples might not significantly differ from that in clinical samples (48). Second, due to limited access to subject resources, we did not recruit individuals with only depression as another control group. It is in generally difficult to accommodate many subject resources in clinical psychology. To our best knowledge, only one study recruited all SAD, SADd, depression, and HC groups but they only tested limited stimulus durations and types (40). Another study also only recruited the SAD, SADd, and HC groups (21). However, without the depression-only group, it is unclear if the observed effects are due to comorbidity or high levels of depression. Thus, the speculation of comorbidity needs to be further verified in future studies. Adding a group with only depression can fill the gap of the current experimental design and will certainly provide a more complete picture of the interaction between anxiety and depression. Third, the dot-probe task based on reaction time was used to investigate attentional bias in this study. This is because (1) the reaction-time-based dot-probe task is easy to implement for special populations, and (2) we aim to examine the effects of subliminal stimuli. Note that the dot-probe task is good for the reaction-time-based experiment, but is different from the classical Posner cueing task used in attention research. In the Posner cueing task, a participant needs to discriminate between two probes rather than respond to one probe as quickly as possible. However, it has been known that this paradigm sometimes provides poor psychometric properties. Several other studies used eye-tracking techniques to investigate attention (15, 49, 50). Eye-tracking methods indeed have better psychometric properties and can reveal more spatiotemporal details of participants' attention deployments. One future direction is to verify our results via eye-tracking tasks. Lastly, ages of the groups were not well-matched. Although we added age as a covariate in statistical analysis and obtain the qualitatively same results, we cannot completely exclude the potential confounding effects of age. In particular, we found that emotion type manifests as a main effect in the younger subjects, indicating more heterogeneous behavioral pattern in younger adults. The exact reason still remains unclear. Future studies should continue to investigate the effect of age on attentional bias.

In conclusion, although wide research interests have been devoted to SAD and depression as independent psychiatric disorders, there is little behavioral evidence for perceptual deficits associated with concurrent SAD and depression. Using the well-established dot-probe paradigm, we find a clear vigilance-avoidance pattern of the SADd group on angry faces and strong attentional preference to disgusted faces in the SAD group. These findings highlight the qualitative difference between comorbid and isolated pathological causes and can potentially be used in future diagnoses to distinguish SAD and depression.

## Data Availability Statement

The original contributions presented in the study are included in the article/supplementary material, further inquiries can be directed to the corresponding author. Requests to access these datasets should be directed to 017110@nankai.edu.cn.

## Ethics Statement

The studies involving human participants were reviewed and approved by the Ethics Committee of Peking University. Written informed consent for participation was not required for this study in accordance with the national legislation and the institutional requirements.

## Author Contributions

TK: design of the study and drafting the paper. XW, NY, and YH: dcquisition of data. R-YZ and ML: revising the paper for important intellectual content. MQ: supervision and funding acquisition. All authors contributed to the article and approved the submitted version.

## Conflict of Interest

The authors declare that the research was conducted in the absence of any commercial or financial relationships that could be construed as a potential conflict of interest.
